# GaN-Based High-Contrast Grating for Refractive Index Sensor Operating Blue–Violet Wavelength Region

**DOI:** 10.3390/s20164444

**Published:** 2020-08-09

**Authors:** Yuusuke Takashima, Masanobu Haraguchi, Yoshiki Naoi

**Affiliations:** 1 Graduate School of Technology and Social Science, Tokushima University, Tokushima 770-8506, Japan; haraguchi.masanobu@tokushima-u.ac.jp (M.H.); naoi@tokushima-u.ac.jp (Y.N.); 2 Institute of Post-LED Photonics, Tokushima University, Tokushima 770-8506, Japan

**Keywords:** refractive index sensor, subwavelength grating, GaN

## Abstract

Owing to its versatility, optical refractive index (RI) sensors with compact size and high chemical stability are very suitable for a wide range of the applications in the internet of things (IoT), such as immunosensor, disease detection, and blood mapping. In this study, a RI sensor with very simple system and high chemical stability was developed using GaN-based high-contrast grating (HCG). The designed HCG pattern was fabricated on GaN-film grown on c-plane sapphire substrate. The fabricated GaN-HCG sensor can detect minuscule RI change of 1.71 × 10^–3^ with extreme simple surface normal irradiation system. The light behavior inside the GaN-HCG was discussed using numerical electromagnetic field calculation, and the deep understand of the sensing mechanism was provided. The simple system and very high chemical stability of our sensor exploit RI sensing applications in IoT society.

## 1. Introduction

A sensing techniques of refractive index (RI) have an important contribution for development of the internet of things (IoT) society, such as engineering, biology, and chemical science [1,2,3]. In particular, the highly sensitive RI sensors which can operate in aqueous possess large benefits because the RI sensing applications are frequently used in aqueous ambient, such as, immunosensor [4], blood cell mapping [5], label-free bio-imaging [6], and disease-detections [7]. The RI sensing applications require high sensitivity with compact optical system and high chemical stability (for example, resistance to oxidation).

The optical RI sensing is one of the most suitable techniques for the applications mentioned above because the sensors do not require the electrical wiring. Many kinds type of RI optical sensing devices have been developed so far using optical resonances in metal surfaces [1,8,9,10,11], nano particles [2,3,12,13,14], nanorings [15,16,17], waveguides [18,19,20,21,22], gratings [23,24,25,26,27,28,29,30], photonic crystals [31,32,33,34], and microstructured optical fibers [35,36,37]. The systems of RI sensing based on surface plasmon resonance (SPR) have been widely used and been commercialized. The SPR sensors utilize the coupling between the light and the free electrons collective oscillation via metal-dielectric interface. Occurring the coupling, the reflectivity of the metal drops at a certain incident angle and wavelength because the input light energy is confined into the metal-dielectric interface. The coupling condition is considerably influenced on RI value around the metal surface, and this induced the shift of the SPR incident angle and wavelength. Hence, SPR sensors are extremely sensitive for minuscule RI changes. However, the momentum compensators, such as prism coupler, are generally essential to occur the SPR, because the momentum of the corrective oscillation of electrons is always larger than that of the incident light [1,9,10,11]. This leads too bulky system for integrated applications.

The RI sensing techniques without the momentum compensators were reported using optical resonances in nano particles [12,13,14], nanorings [15,16,17], waveguides [18,19,20,21,22], gratings [23,24,25,26,27,28,29,30], photonic crystals [31,32,33,34], and microstructured optical fibers [35,36,37]. The sensors can detect RI change of the ambient around the resonators without bulky compensators. The no usage of the compensators is very beneficial for the integrated applications of RI sensing owing to its compactness. Very recently, high-contrast grating (HCG) based biosensor with extremely simple excitation system, high sensitivity, and rapid response time have been reported [38,39]. The HCG sensors were composed of Si subwavelength grating fully surrounded by low RI materials (SiO_2_ and air). The abrupt periodic RI contrast between the Si grating and the surrounding materials supports not only fundamental optical eigenmode but also the higher-orders inside the HCG [40,41,42]. The excited eigenmodes are orthogonal in HCG, while the modes couple each other and form the interference at the top and bottom boundaries of HCG. Depending the phase difference between the modes, the destructive or constructive interferences, which induces resonant reflection (or transmission) peak (or dip), are formed. The minuscule RI variation by surface binding a biomolecule causes the deviation of the mode’s phase relation. Using the interference between the fundamental and the higher-order modes in the HCG, T. Sun et al. experimentally realized 100 pg/mL cardiac disease detection within 4 min [38]. However, the chemical robustness of the Si based nanostructures are not high enough.

In this work, we tried to develop an RI sensor operating blue–violet wavelength using monolithic HCG composed of GaN, which has high RI value and high chemical stability. In addition to high chemical stability, our sensor has the following unique advantages. (1) Blue–violet wavelength operation: the blue–violet light is hardly absorbed in water ambient, and the sensor is very suitable for the RI sensing applications because the applications are often used in aqueous ambient (RI value is around 1.33). (2) Compatibility with light source: GaN is the most widely used as material for the commercialized Light-Emitting Diodes (LEDs) visible and ultraviolet wavelengths. Our GaN-HCG RI sensor can directly be incorporated onto the surface of LEDs whereas the others cannot. This is very useful for compact and portable devices because our sensor and light source can be integrated. Thus, it is very useful and novel to measure the RI around 1.33 with our GaN-HCG sensor for the RI sensing applications. The GaN-film was grown on c-sapphire substrate using metal organic chemical vaper deposition (MOCVD) method, and the HCG pattern was formed by electron beam (EB) lithography and inductive coupled plasma (ICP) etching techniques. The peak at blue–violet wavelength of 405 nm was obtained in the reflection spectrum of the fabricated GaN-HCG. The reflection peak intensity significantly decreased when the RI value of ambient slightly increased. We also discussed and revealed the mechanism of RI detection of our sensor using electromagnetic field distribution calculation by finite-dereference time-domain (FDTD) method.

## 2. Operation for RI Sensing

**Figure 1:**
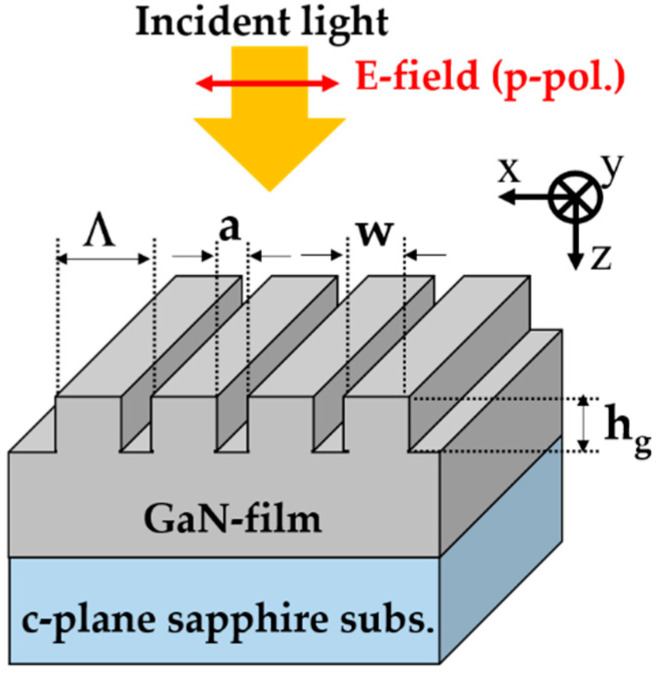
Schematic diagram of the monolithic GaN- high-contrast grating (HCG) on *c*-plane sapphire substrate.

In this section, we described the basic rule of RI sensing technique with HCG. Figure 1 illustrates the schematic of the monolithic GaN-HCG geometry. The GaN-HCG with the shorter period than the incident wavelength λ is monolithically arranged on the top of the GaN-film on *c*-plane sapphire substrate. The incident plane wave is irradiated normally to the HCG. The electric field of the incident light is p-polarized (red arrow direction in Figure 1). The grating period, grating-bar width, grating thickness, and air-gap width are represented as symbol Λ, w, h_g_, and a, respectively.

Outside HCG, the subwavelength period permits to cause no diffractions except 0th orders [43]. On the other hand, the periodic RI distribution of the HCG introduces optical eigenmodes, which have quite different field profiles from outside one. When the RI value of the grating possess large abrupt difference to that of the surrounding material, not only fundamental mode (namely, the lowest-order) but also the higher-orders can couple with the surface normal irradiation [40,41,42]. These excited eigenmodes are orthogonal each other and propagate separately the along the grating. These modes couple and mix with each other via the top and bottom boundaries of the HCG. If the field amplitudes in the grating-bars and the air-gaps are anti-phase at the boundary between the HCG and the GaN-film, the spatial field average approaches to zero. This destructive interference weakens the transmitted field through the HCG and causes resonant reflection at the boundary. As a result, one can see peak in its reflection spectrum of the HCG. The intensity of the resonant reflection peak is affected by the RI value of the medium around the HCG because the amplitudes and the phases of each eigenmodes strongly depend on the RI contrast between the HCG and the medium. Hence, we can measure the slightly change of RI value of the surrounding medium. The our detection method based on the intensity at a certain wavelength is useful for integrated devices because it does not require a broadband light sources and can be incorporated onto the surface of narrowband solid light source, such as LED.

Based on the operation rule of HCG, we tried to design the structural geometry of the GaN-HCG. In order to obtain a reflection peak, it is necessary that the excited eigenmodes destructively interfere each other to become zero average field at the interface between HCG and GaN-film. Based on the criteria, the structural parameters of the GaN-HCG were selected using the wavenumber dispersion relation of the HCG eigenmodes. In the HCG, the wavenumber dispersion relation is given by [40,41,42]:

(1) nbar−2kwtan(kww2)=−katan(kaa2)

where, the term of n_bar_, k_w_, and k_a_, correspond to the RI value of HCG, eigenmode’s lateral (*x*-direction) wavenumber in the bars, and the air-gaps, respectively. In our case, the RI value of GaN was employed as *n*_bar_ [44]. In addition, the relation between the lateral wavenumbers *k*_w_, *k*_a_, and the longitudinal wavenumber β (namely, propagation constant for *z*-direction) is also found as follows [40,41,42].

(2) β2=(2πλ)2−ka2=(2πnbarλ)2−kw2

According to Equations (1) and (2), we can find that the RI of grating, period and filling factor determine the eigenmode’s propagation constant β. The grating thickness also defines the mode phase when the eigenmode with β propagates from the top to the bottom of the HCG, and has large influence on the interference between the eigenmodes. Based on the wavenumber relations, we designed the GaN-HCG, and the parameters set to Λ = 200 nm, w = 180 nm, a = 20 nm, and h_g_ = 250 nm, respectively, in order to form the destructive interference blue–violet wavelength region.

## 3. Experimental Procedure

### 3.1. Fabrication of GaN High-Contrast Grating

**Figure 2:**
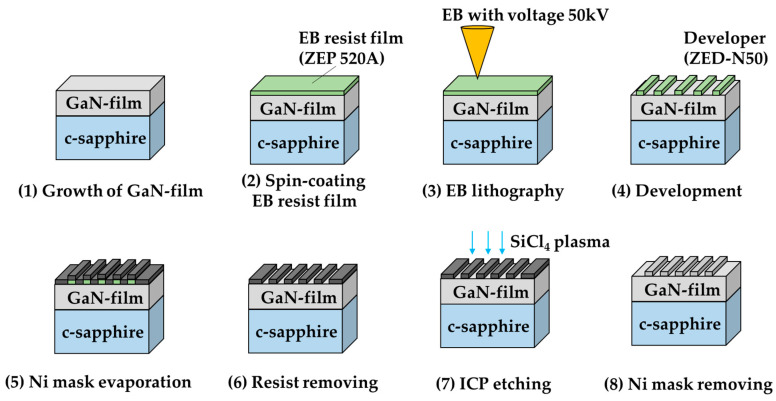
Fabrication protocol of the GaN-HCG.

In this section, we describe the fabrication protocol of the GaN-HCG. The protocol is shown in Figure 2.

**Figure 3:**
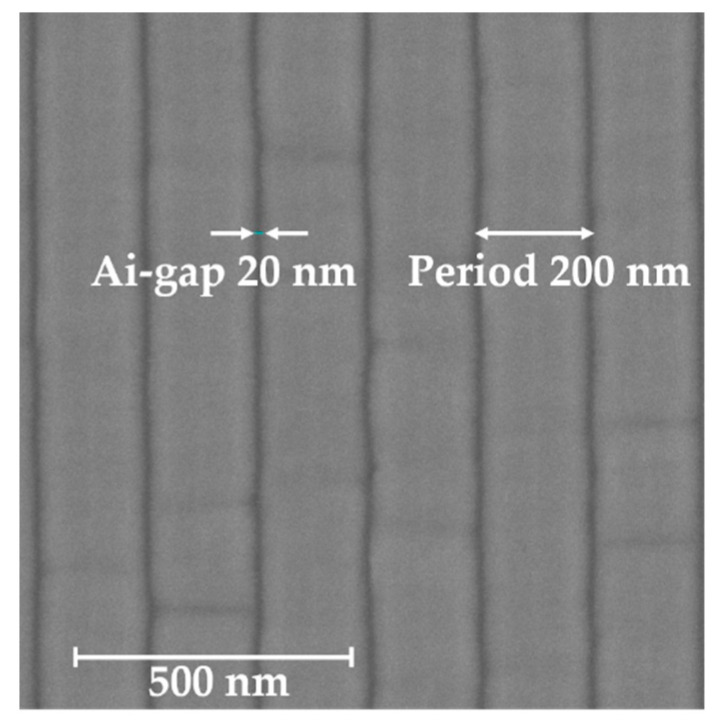
Scanning electron microscope (SEM) image of the GaN-HCG.

A GaN-film was grown on the c-plane sapphire substrate by using MOCVD technique. According to the reflection spectrum of thin-film interference, the thickness of the grown GaN-film is about 6 μm. The pattern of the designed HCG was developed via lithography technique with EB. The HCG fabrication process flow is as follows. The surface of the GaN sample was coated with about 100 nm thickness EB resist-film (ZEP520A: Zeon Co., Tokyo, Japan) using spin-coating method. The spin conditions of 500 and 3000 rpm were employed for 5 s and 90 s, respectively. After coating, the sample was baked at 120 °C for 30 min. The EB with acceleration voltage of 50 kV was irradiated in the vacuum chamber with 2 × 10^−5^ Pa in order to draw the HCG pattern. The developer (ZED-N50: Zeon Co., Tokyo, Japan) was used to form the EB resist grating pattern. Next, a 50 nm Ni metal mask for ICP etching was evaporated onto the patterned sample with 2.6 × 10^−4^ Pa pressure, and the patterned resist-film was removed. We transcribed the Ni mask pattern onto the GaN-film using ICP etching technique. The sample was etched by SiCl_4_ plasma gas with ICP power of 100 W and 50 V bias for 35 min. The SiCl_4_ flow rate and the pressure during etching process were 4 sccm and 0.5 Pa, respectively. Finally, we removed the Ni mask by dilute nitric acid. The scanning electron microscope (SEM) image of the fabricated GaN-HCG is shown in Figure 3. The scale bar in Figure 3 corresponds to 500 nm. The grating period and the air-gap are 200 nm and 20 nm, respectively.

### 3.2. Optical Irradiation System

**Figure 4:**
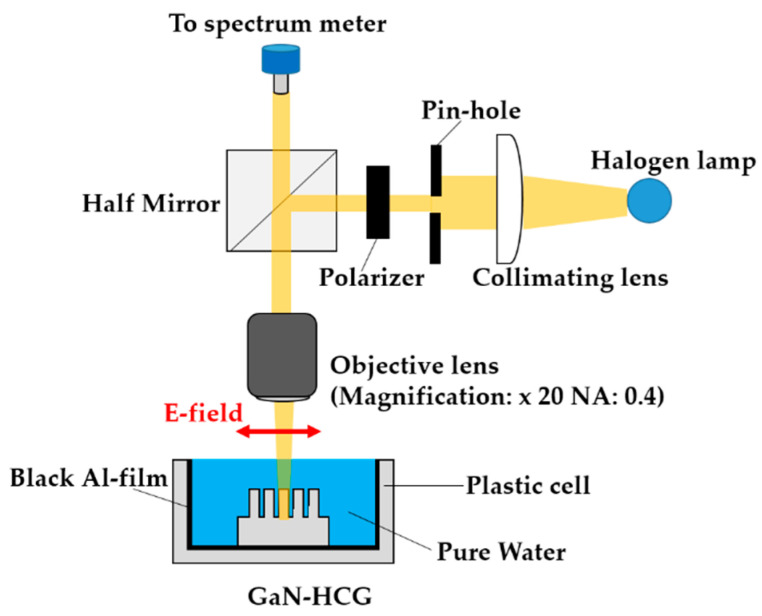
Optical irradiation system for characterizing optical properties and evaluating refractive index (RI) sensing performance of our sensor.

Figure 4 illustrates the diagram of optical irradiation system for characterizing optical properties and evaluating RI sensing performance of the fabricated GaN-HCG. The fabricated sample was placed on the plastic cell. The plastic cell was covered by blacked Al film to prevent the reflection at the surface of the cell. The cell was fulfilled with pure water. The light of the halogen lamp passed through the pin-hole to irradiate only HCG region. The electric field of light was controlled by polarizer, and the light was p-polarized (the electric field direction was perpendicular to the grating fingers). The p-polarized light was focused and irradiated normally to the GaN-HCG using the objective lens (magnification: 20×, Numerical aperture: 0.4), and the reflection spectrum of the HCG was measured by spectrometer (OP-Flame-S Package: Ocean Photonics Co., Tokyo, Japan). We added ethanol to pure water in the plastic cell. With increasing the ethanol concentration, the RI value of the ambient around the sample slightly varied. We investigated the dependence of the reflection spectrum on the RI value of the ambient around the sample. We made 10 measurements at each ethanol concentrations. The measurements were also in random order of ethanol concentrations.

The RI values of the solution were measured by commercialized refractometer (RAB-18: As One Co., Osaka, Japan). The detail of the measurement of RI value is as follows. We prepared 6 ethanol solutions with different concentrations. The solutions were titrated onto the prism of the refractometer, and the internal-total-reflection between the prism and the solutions was used for defining the RI value of the solutions. The 10 measurements were done for each ethanol concentrations.

### 3.3. FDTD Field Calculation Model and Calculation Conditions

**Figure 5:**
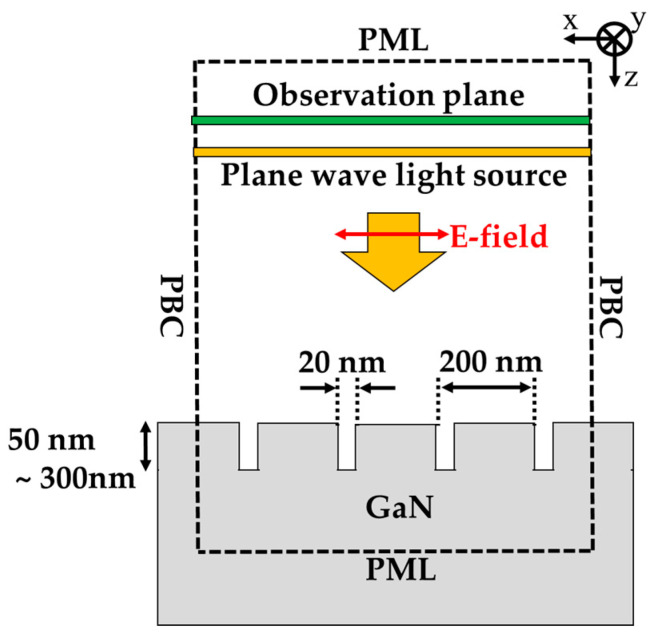
Two dimensional- finite-dereference time-domain (FDTD) calculation model of the GaN-HCG.

The diagram of the calculation model is shown in Figure 5. The components in the calculation model has infinite length for y-direction because the length of the fabricated structure is 300 μm, which is much larger than the incident wavelength. The calculation region of *x*-*z* plane is illustrated by dashed square. The size of the calculation area for *x*- and *z*-direction were 800 and 1710 nm, respectively. The periodic boundary condition (PBC), where the fields in the calculation region were infinitely repeating, was used as both x-direction calculation boundaries. The semi-infinite thickness GaN-film under HCG were assumed to remove the effect of GaN thin-film interference for understanding of the light behavior in the HCG. Thus, the perfect matched layer (PML) boundary condition, which perfectly absorbs the light outside the boundary, was used for z-directions. The yellow square indicates light source, and the incident plane wave was normally irradiated to the GaN-HCG. Only Poynting vector of the reflected light by the HCG was evaluated at observation plane placed above the light source. The spatial grid size and time interval in the calculation were 2 × 2 nm^2^ and 4.67 × 10^−18^ s, respectively. The detail for the calculation method are represented in our previous paper [45]. The GaN-HCG with Λ = 200 nm, w = 180 nm, and h_g_ = 50 to 300 nm was monolithically arranged on the GaN-film.

## 4. Results and Discussions

**Figure 6:**
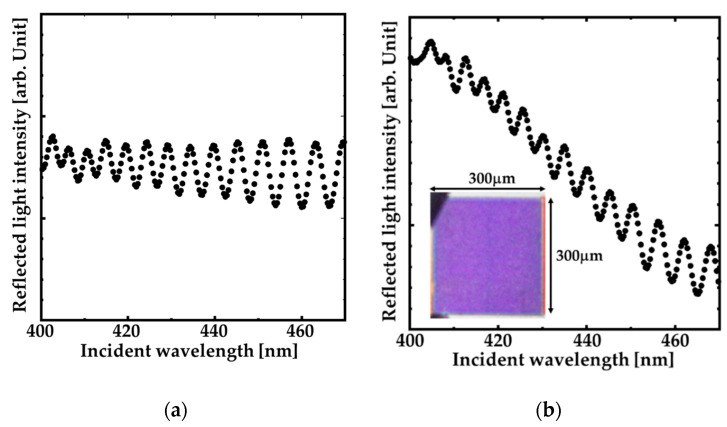
Normal reflection spectrum of: (**a**) GaN-film and (**b**) the fabricated GaN-HCG. The inset shows the optical microscope image of the GaN-HCG region.

Figure 6a,b show the experimental reflection spectrum of the GaN-film and the fabricated GaN-HCG surrounded by pure water under p-polarized light illumination. The reflected intensities were evaluated by that of Al reference mirror. The nano structures in these reflection spectra were originated from the thin-film interference of GaN-film. As shown in Figure 6a, the spectrum of the GaN-film shows no reflection peak. On the other hand, the peak around the wavelength of 405 nm was obtained at GaN-HCG reflection spectrum. The inset of Figure 6b is optical microscope image of the GaN-HCG region. The 300 μm × 300 μm region of the GaN-HCG is colored with blue–violet, and the image corresponds to the HCG reflection spectrum.

**Figure 7:**
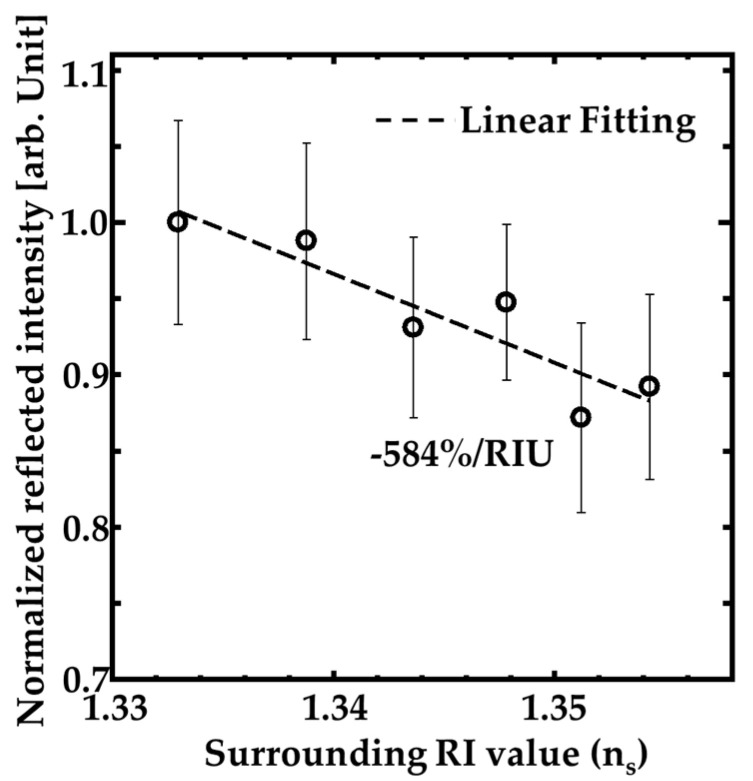
Dependence of the reflection peak intensity at the wavelength of 405 nm on the surrounded RI value around the HCG.

Moreover, we investigated the performance of the GaN-HCG sensor. Figure 7 indicates the dependence of the HCG reflection peak intensity on the RI value around the sample (n_s_). The measurements for the dependence of the intensity on n_s_ were done in random order of n_s_. The open circles and ranges of the error bars mean medians and ± standard deviation ranges over 10 measurements. The intensity at the reflection peak decreased with slightly increasing n_s_ value. The dashed line in Figure 7 is linear fitting by least-squares method, and the intensity decreasing inclination reached to 584% per refractive index unit (RIU). Assuming that 1% intensity change can be detected, the sensitivity indicates that the RI resolution of our sensor is RI of 1.71 × 10^−3^. These results mean our GaN-HCG sensor demonstrated detection of the slight RI change in spite of very simple surface normal irradiation system.

**Figure 8:**
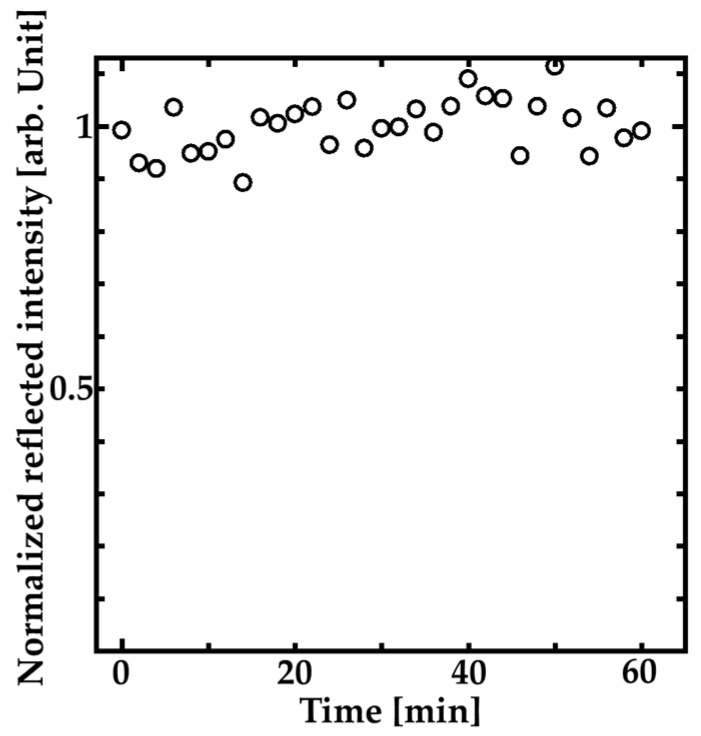
Time dependence of the reflection peak intensity of the fabricated HCG surrounded pure water.

We also investigated the time stability of the reflection peak intensity. Figure 8 shows the time dependence of the reflection peak intensity of the HCG surrounded by pure water. The intensities were normalized by the average intensity value during the measurement. The intensities were measured each 2 min during 1 h. The standard deviation of the intensity fluctuation during 1 h is about ±0.05. The result means that the time stability of our sensor is enough for RI measurement because the fluctuation is smaller than the experimental error bars in Figure 7.

To discuss and interpret the light behavior in GaN-HCG, we investigated the electromagnetic field distribution of the grating by using FDTD calculation method. The detail of the calculation model and the calculation conditions are described in Section 3.3.

**Figure 9:**
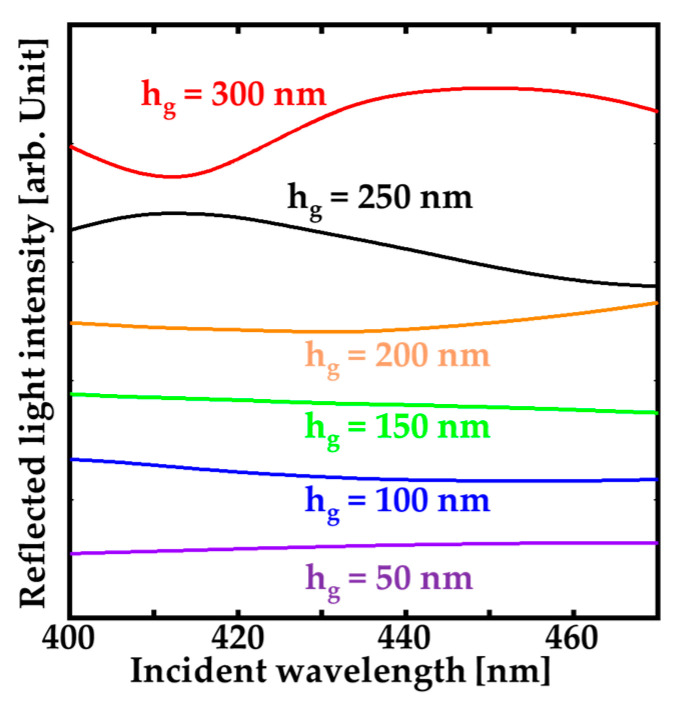
FDTD calculated reflection spectrum of GaN-HCG with Λ = 200, w = 180 nm, and h_g_ = 50 to 300 nm.

For various grating thickness h_g_, the normal reflection spectra of the GaN-HCG fixed to Λ = 200 and w =180 nm were calculated in Figure 9. These structural parameters of Λ and w were taken in SEM image of the fabricated HCG (Figure 3). The spectra of the GaN-HCG strongly depends on h_g_ because h_g_ determines the phases of the each modes in the interference, as described in Section 2. Reflection peak cannot be obtained at h_g_ = 50 nm to 200 nm because the phase differences between the excited modes were small due to the thin grating thickness. For h_g_ = 250 and 300 nm, we can see the peak in the spectrum at the wavelength of 415 and 450 nm, respectively. According to calculation spectra, the actual etching depth of the fabricated GaN-HCG is estimated to be about 250 nm because the calculated spectrum shows good agreement of the experimental results.

**Figure 10:**
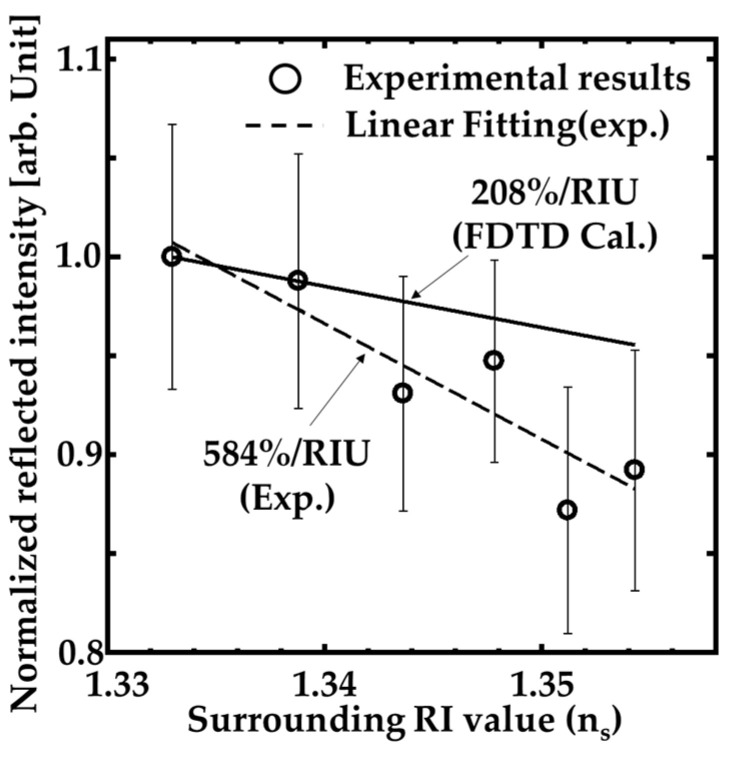
Dependence of the peak intensity of GaN-HCG on n_s_ (FDTD calculation).

We calculated RI sensing performance of the GaN-HCG with h_g_ = 250 nm in shown in Figure 10. The solid line Figure 10 also shows the calculated dependence of the peak intensity of GaN-HCG reflection spectrum on n_s_. The open circles and dashed line are experimental results and linear fitting, respectively. The calculated peak intensity decreased with the slight increasing of n_s_ value. The calculated intensity change is 208% per RIU.

**Figure 11:**
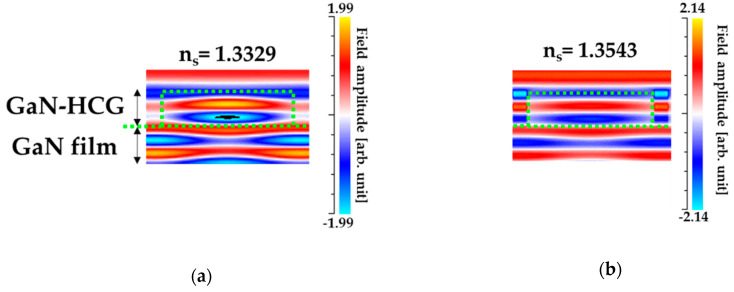
The calculated magnetic field profile at the wavelength of 415 nm with (**a**) n_s_ = 1.3329 (water) (**b**) n_s_ = 1.3543. The amplitudes were normalized by that of the incident light.

Also, the magnetic field distributions around the GaN-HCG were illustrated in Figure 11a,b. The field amplitudes were normalized by that incident light. Inside the GaN-HCG, the field profile was quite difference from that of plane wave. In the case of n_s_ = 1.3329 (Figure 11a), the field concentrates into grating-bar, and the field in the grating-bar is out-phase with that in the air-gap at the bottom boundary between HCG and GaN-film. This fact corresponds that the input light was resonantly reflected at the HCG bottom boundary, and the peak can be resonantly obtained in the reflection spectrum [40,41,42]. When the RI value n_s_ varied to 1.3543 (Figure 11b), the field evenly distributed at the bar and air-gap. The phase relation between the bar and air-gap is in-phase, and the interference between modes is also significantly influenced. This can be explained as follows. The increasing of n_s_ has influence on the phase and amplitude of all excited modes. The effect of increasing n_s_ in each modes accumulated to the interference. Thus, the significant intensity change of the reflection peak can be obtained for the slight increasing n_s_.

Comparing the calculation results with the experimental one, the experimental sensitivity is higher than the calculated one, as shown in Figure 10. The cause of the higher sensitivity can be attributed to the fabrication imperfectness of the actual GaN-HCG. The fabricated HCG includes the fluctuation of the structural geometry, such as rough side walls of the HCG (See SEM image in Figure 3) whereas ideal shape of the grating was assumed in the FDTD simulation. When the grating shape is not ideal rectangle and rough, the effective RI of the HCG bar is locally reduced. This causes nonuniformity of the mode’s phases, and the interference of the modes at the HCG and GaN-film interface also become more unstable for the change of n_s_. This consideration opens up the new design method for high sensitivity of RI sensing with HCG, and the HCG will be optimized for more high sensitivity in further work.

Finally, our sensor performance is compared with other reports. The comparison is shown in Table 1. As shown in Table 1, the sensitivity of our sensor with very simple normal incidence achieved comparable sensitivity of other sensor [30]. Our sensor also operates at blue–violet wavelength (405 nm). This is very suitable for applications used in aqueous ambient, such as biosensors, because the light absorption of the water at blue–violet wavelength is very lower than that at other wavelengths [46]. Moreover, the extremely high stability of GaN-HCG sensor provides the chemical robustness for several corrosions, such as oxidation reaction. Its extreme simple optical system and high chemical stability of our sensor are of great use for the portable applications of RI sensing in IoT society. Also, our GaN-HCG can be directly incorporated GaN-based LED. In our further work, the hybrid device with integrated RI sensor and GaN-based LED will be reported in the elsewhere.

## Figures and Tables

**Table 1:** Comparison of several RI sensor performance.

Sensor Description	RI Range	RI Detection Limit (RIU)	Optical Setups	Operating Wavelength (nm)	Sensor Material
SPR based optical fiber [8]	1.33–1.40	2 × 10^−4^	Optical fiber	670	Au
Flexible fiber-optic SPR [10]	1.33–1.3305	3.672 × 10^−5^	Optical fiber and prism	1550	Ag
Subwavelength grating waveguide micro-ring resonator [20]	1.333–1.343	5.4645 × 10^−^^5^	On chip	1550	Si_3_N_4_
Guided-mode-resonance system with optofluidic [21]	1.333–1.373	4.10 × 10^−^^5^	Oblique incidence	525	TiO_2_
Chirped guided-mode resonance grating [25]	1.3324–1.3444	2.37 × 10^−^^4^	Normal incidence	840	Si_3_N_4_
Gradient period guided mode resonance grating [30]	1.333–1.442	5.58 × 10^−^^3^	Normal incidence	640	TiO_2_
Photonic crystal heterostructure cavity [32]	1.3149–1.3392	7.8 × 10^−^^6^	On chip	1550	Si
Multi-plasmon resonance in microstructured optical fiber [35]	1.33–1.39	1.0 × 10^−^^5^	Optical fiber	500–900	Au
Localized SPR in photonic crystal fiber [36]	1.33–1.43	9 ×10^−^^7^	Optical fiber	600–1100	Au
Long-range SPR in H-shaped optical fiber [37]	1.33–1.39	1.3 × 10^−^^5^	Optical fiber	800–1300	Au
This work	1.333–1.354	1.71 × 10^−3^	Normal incidence	405	GaN
